# SeA–SuA Eutectic Phase Change Materials with PDMS-Modified Graphene Oxide for Medium-Temperature Thermal Energy Storage

**DOI:** 10.3390/ma19214600

**Published:** 2026-10-30

**Authors:** Yanyu Zhao, Xiaozhao Li, Zhiming Xu, Xue Wang, Huiyuan Wang, Peng Zhao

**Affiliations:** 1 State Key Laboratory of Intelligent Construction and Healthy Operation and Maintenance of Deep Underground Engineering, China University of Mining and Technology, Xuzhou 221116, China; 2 Yunlong Lake Laboratory of Deep Underground Science and Engineering, Xuzhou 221116, China

**Keywords:** eutectic phase change material, interfacial engineering, thermal energy storage, thermal conductivity enhancement, dispersion stability

## Abstract

Phase change materials are promising for medium-temperature thermal energy storage due to their high latent heat storage capacity. However, their applications are limited by low thermal conductivity and the trade-off between thermal conductivity enhancement and latent heat retention when conductive fillers are introduced. In this study, polydimethylsiloxane-modified graphene oxide (MGO) was incorporated into a sebacic acid–succinic acid (SeA–SuA) eutectic PCM to improve filler dispersion and filler–matrix contact while maintaining thermal storage performance. At an MGO loading of 0.25 wt%, the composite exhibited a melting peak temperature of 128.9 °C, a melting enthalpy of 234.8 J g^−1^, and a room-temperature thermal conductivity of 0.499 W m^−1^ K^−1^. Compared with the composite containing unmodified graphene oxide (GO) at the same loading, the MGO-based PCM exhibited better latent heat retention and higher thermal conductivity, which may be associated with reduced filler aggregation and improved filler–matrix contact. After 800 accelerated thermal cycles, the composite retained 95.2% of its initial melting enthalpy and 87.8% of its initial thermal conductivity. These results demonstrate that combining eutectic composition regulation with GO surface modification is a feasible strategy for achieving balanced thermal storage, transport, and cycling performance in medium-temperature composite PCMs.

## 1. Introduction

The increasing integration of renewable energy into energy systems has increased the demand for thermal energy storage (TES) technologies to mitigate mismatches between energy supply and demand [1]. Among various TES technologies, Carnot batteries have attracted increasing attention because of their potential cost advantages and reduced dependence on specific geographical conditions [2,3,4]. In typical Carnot battery systems employing heat pump–organic Rankine cycle (HP–ORC) configurations, electrical energy is converted into thermal energy and stored in TES units during charging, while the stored thermal energy is converted back into electricity during discharging [5]. The selection of TES materials plays a critical role in determining the thermal storage capacity, charging/discharging efficiency, and long-term operational reliability of such systems. For Carnot battery systems coupled with low-grade heat sources, the required storage temperature can fall within the medium-temperature range of approximately 100–150 °C, depending on the characteristics of the heat source and working fluid [6]. Therefore, developing TES materials with suitable phase transition temperatures, high thermal conductivity, excellent thermal stability, and reliable cycling performance is essential for achieving efficient medium-temperature thermal energy storage.

Phase change materials (PCMs) are regarded as promising TES materials owing to their high latent heat storage capacity and reversible phase transition characteristics [7]. Among various PCMs, organic PCMs have received extensive attention because of their favorable chemical stability, low corrosiveness, and potential economic advantages. However, the practical application of single-component organic PCMs is still limited by their fixed phase transition temperatures and intrinsically poor heat transfer capability. On the one hand, the fixed phase transition temperature of individual organic PCMs limits their applicability under different operating conditions [8]. On the other hand, their low thermal conductivity results in slow thermal response and limits the charging/discharging rates of TES systems [9]. Previous studies on cold thermal energy storage have highlighted low thermal conductivity, supercooling, and phase separation as key limitations of PCMs [10,11]. Composition adjustment has also been explored to regulate phase-change temperatures, although changes in latent heat and supercooling must be considered [12]. Despite the differences in operating temperature ranges, these studies underscore the importance of balancing temperature regulation, thermal transport, and cycling stability in PCM design. For medium-temperature TES applications, binary or multicomponent eutectic systems have been widely explored to tailor the phase transition behavior of organic PCMs. By optimizing the composition of eutectic mixtures, the phase transition temperature can be adjusted while maintaining high latent heat storage capacity, providing greater flexibility in designing PCM-based TES materials for different application conditions [13,14,15].

Succinic acid (SuA) possesses a relatively high latent heat of fusion (approximately 295 J g^−1^) and a melting temperature of around 186 °C, making it a potential organic PCM for thermal energy storage. However, under certain testing conditions, the reported onset of thermal decomposition lies relatively close to its melting point, suggesting a limited thermal stability window in the molten state [16]. In addition, its phase transition temperature is higher than the target range considered for medium-temperature TES in this work. Previous studies have explored SuA-based multicomponent thermal storage systems. For example, Saher et al. reported a boric acid–SuA eutectic system with a reversible storage capacity of up to 394 J g^−1^ through the combined contributions of sensible, latent, and thermochemical heat storage [17]. However, its reversible performance relies on retaining the water generated during boric acid dehydration, imposing additional requirements on system sealing and moisture retention. In contrast, sebacic acid (SeA) exhibits a lower phase transition temperature of approximately 133–136 °C and a latent heat of about 243 J g^−1^, making it suitable for medium-temperature thermal energy storage applications [18,19]. Nevertheless, its fixed phase transition temperature limits temperature matching across different operating conditions. Therefore, combining SeA and SuA into a binary eutectic system provides a rational approach for tailoring the phase transition temperature while maintaining relatively high latent heat storage capacity, thereby improving temperature matching and reducing the need for operation at the higher temperatures required for pure SuA.

Although eutectic formulation enables tailoring of the phase transition temperature, it does not overcome the intrinsically low thermal conductivity of organic PCMs, which remains a major limitation to rapid thermal charging and discharging [20]. Incorporating thermally conductive fillers is a widely adopted approach to enhance thermal transport in composite PCMs. Typical fillers include ceramic materials such as aluminum nitride, alumina, and hexagonal boron nitride [21,22,23], carbon-based materials such as carbon nanotubes, graphene, and expanded graphite [24,25,26], and metallic fillers such as silver, aluminum, and copper [27,28,29]. These fillers can provide additional thermal conduction pathways and thereby improve the effective thermal conductivity and thermal response of PCM composites. However, most conventional conductive fillers do not participate in the phase transition within the target operating temperature range. Their incorporation therefore reduces the PCM mass fraction and may also alter the crystallization behavior of the matrix, resulting in a trade-off between thermal conductivity enhancement and latent heat retention [30]. Accordingly, enhancing thermal transport at low filler loadings while minimizing latent heat loss remains a key challenge in the design of composite PCMs.

Graphene oxide (GO), owing to its two-dimensional sheet-like structure, has been widely investigated as a thermally conductive filler for organic PCMs [31]. In addition, the hydroxyl, epoxy, carbonyl, and carboxyl groups distributed on its basal planes and edges provide reactive sites for further surface modification [32]. However, GO sheets are prone to aggregation and nonuniform dispersion within PCM matrices, which can reduce effective filler–matrix contact and limit the formation of continuous heat-transfer pathways [33]. Surface modification of GO therefore represents a potential strategy for improving filler dispersion and regulating filler–matrix interactions in organic PCM composites. Polydimethylsiloxane (PDMS), with its flexible Si–O–Si backbone and hydrophobic methyl groups, may alter the surface characteristics of GO and suppress sheet restacking through steric effects, thereby modifying the interactions between the filler and organic matrix. Nevertheless, the dispersion behavior of PDMS-modified GO (MGO) in the SeA–SuA eutectic matrix and its effects on both thermal transport and latent heat retention remain unclear. A systematic evaluation is therefore required to determine whether PDMS modification can improve filler dispersion and filler–matrix contact while maintaining the thermal storage capability of the eutectic PCM.

**Figure 1:**
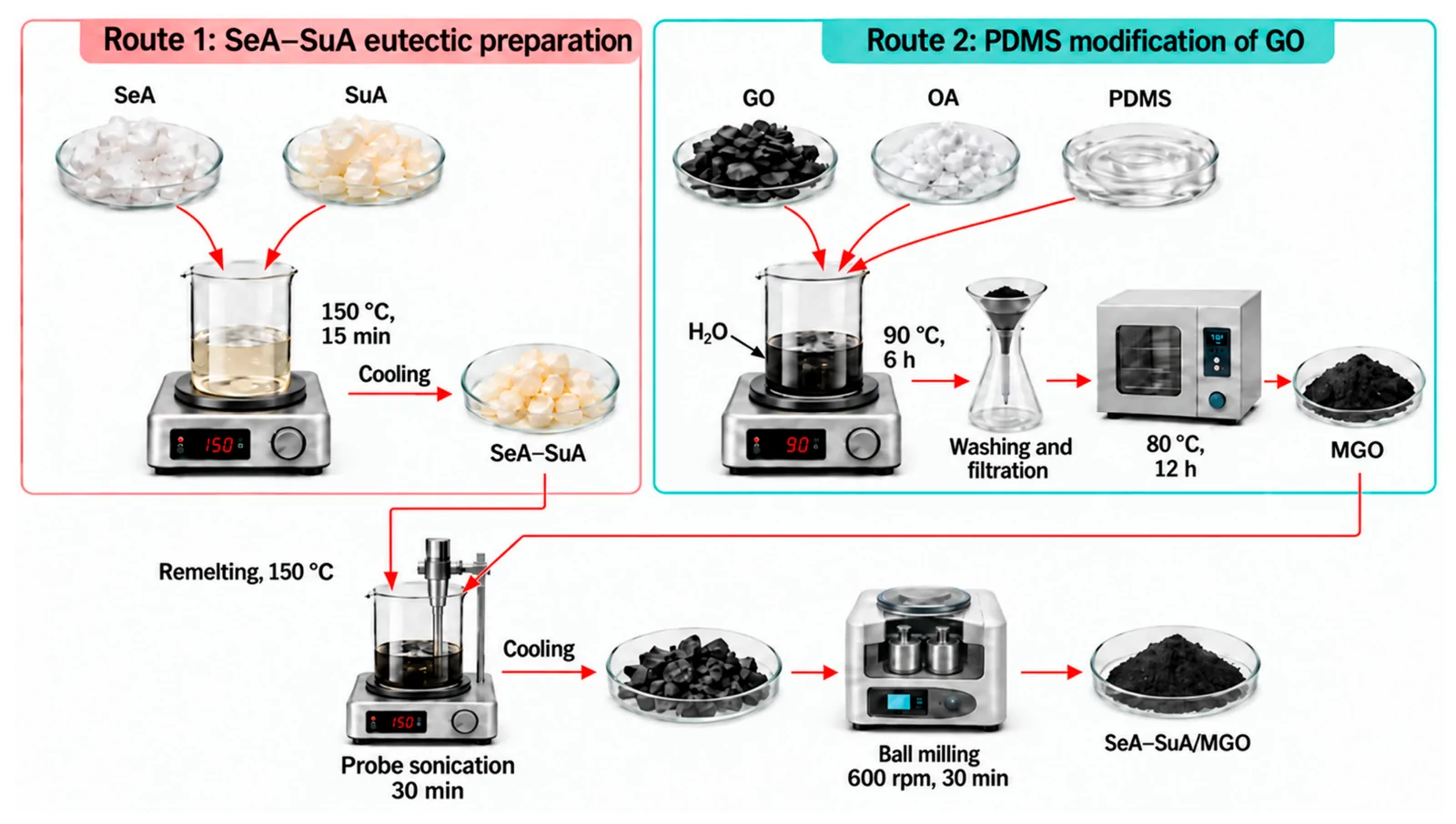
Preparation of the SeA–SuA/MGO composite PCM. The schematic was generated using ChatGPT Images 2.5 (OpenAI, San Francisco, CA, USA) based on an original author-drawn sketch and iteratively refined through author-directed adjustments to the layout and labels.

Based on the above considerations, this study combines eutectic composition regulation with PDMS modification of GO to develop a SeA–SuA/MGO composite PCM for medium-temperature thermal energy storage, as schematically illustrated in Figure 1. The SeA–SuA eutectic without conductive filler was used as the baseline, while a composite containing 0.25 wt% unmodified GO was prepared as a control to assess the effect of PDMS surface modification at the same nominal filler loading. This comparison enables the effects of conductive filler incorporation and PDMS modification to be evaluated separately against the eutectic baseline. A combination of complementary characterization techniques and thermophysical measurements was employed to investigate the surface chemistry and morphology of the fillers, their dispersion within the eutectic matrix, and the phase-change behavior, thermal stability, and thermal transport performance of the resulting PCM systems. In addition, thermal cycling and macroscopic dispersion tests were conducted to evaluate performance retention and dispersion stability during repeated melting and solidification. The macroscopic behavior of GO- and MGO-containing composites under identical cycling conditions was further compared to assess phase separation and filler sedimentation in the molten state. This study aims to investigate how eutectic composition regulation and PDMS surface modification of GO affect the thermal storage and thermal transport performance of the composite PCM and to evaluate whether improved filler dispersion and filler–matrix contact can enhance thermal transport while maintaining high latent heat storage capacity.

## 2. Materials and Methods

### 2.1. Materials

Sebacic acid (SeA, 99%) was purchased from Sinopharm Chemical Reagent Co., Ltd. (Shanghai, China). Graphene oxide (GO), with a supplier-reported sheet thickness of approximately 1 nm and a lateral size of 0.2–10 μm, was purchased from Shanghai Macklin Biochemical Technology Co., Ltd. (Shanghai, China). Succinic acid (SuA, 99%), oxalic acid dihydrate (OA, 99%), and hydroxyl-terminated polydimethylsiloxane (PDMS, nominal molecular weight of approximately 2000) were purchased from Shanghai Aladdin Biochemical Technology Co., Ltd. (Shanghai, China). Deionized water was used for GO modification and subsequent washing procedures.

### 2.2. Preparation of the SeA–SuA Eutectic PCM

The theoretical eutectic composition of the SeA–SuA binary system was predicted using the Schrader equation, yielding a SeA:SuA molar ratio of 0.75:0.25. SeA and SuA were weighed according to this molar ratio and placed in a beaker. The mixture was heated to 150 °C under continuous stirring until a homogeneous melt was formed. The melt was then stirred at 150 °C for an additional 15 min to ensure uniform mixing. The melt was then cooled to room temperature and allowed to solidify completely. The obtained SeA–SuA PCM at the predicted eutectic composition was used for subsequent composite preparation and characterization.

### 2.3. Preparation of PDMS-Modified GO

GO (1 g), OA (3 g), and PDMS (3 g) were dispersed in 200 mL of deionized water, and the resulting suspension was continuously stirred at 90 °C for 6 h. After cooling to room temperature, the suspension was filtered, and the recovered solid was thoroughly washed with deionized water to remove residual reagents. The recovered solid was subsequently dried in an oven at 80 °C for 12 h. The resulting PDMS-modified GO (MGO) was used for subsequent composite PCM preparation.

### 2.4. Preparation of SeA–SuA/GO and SeA–SuA/MGO Composite PCMs

The SeA–SuA eutectic PCM was remelted at 150 °C until fully molten. GO or MGO was then separately added at a nominal loading of 0.25 wt% to prepare SeA–SuA/GO and SeA–SuA/MGO, respectively. The mixtures were probe-sonicated at 300 W and 20 kHz for 30 min while being maintained at 150 °C. After sonication, the samples were cooled to room temperature and allowed to solidify completely. The solidified composites were then ball-milled at 600 rpm for 30 min to pulverize and homogenize the samples. The GO- and MGO-containing composites were designated 0.25GO and 0.25MGO, respectively, while the filler-free eutectic PCM was designated SeA–SuA and used as the baseline. The nominal compositions of the samples are summarized in Table 1.

### 2.5. Characterization

Phase-change behavior was characterized by differential scanning calorimetry (DSC; SETLINE DSC, SETARAM, Caluire-et-Cuire, France) at a heating and cooling rate of 5 °C min^−1^. The crystalline structures of the pristine components and the SeA–SuA eutectic PCM were examined by X-ray diffraction (XRD; D8 ADVANCE, Bruker, Germany) using Cu Kα radiation (λ = 0.15418 nm) over a 2θ range of 4–70°. Fourier-transform infrared spectroscopy (FTIR; VERTEX 80v, Bruker, Germany) and X-ray photoelectron spectroscopy (XPS; ESCALAB 250Xi, Thermo Fisher Scientific, Waltham, MA, USA) were employed to investigate changes in GO surface functional groups and elemental composition after PDMS modification. The morphologies of GO, MGO, and the composite PCMs were examined using field-emission scanning electron microscopy (FE-SEM; GAIA3 XMH, TESCAN, Brno, Czech Republic), while energy-dispersive X-ray spectroscopy (EDS) was used to analyze the elemental distribution in MGO. Thermal conductivity was measured at room temperature using the transient plane source method with a thermal constants analyzer (TPS 2500 S, Hot Disk AB, Göteborg, Sweden) on pelletized samples. Thermogravimetric analysis (TGA; STA 2500, NETZSCH, Selb, Germany) was performed to evaluate the thermal stability of SeA–SuA and its composite PCMs by heating the samples to 800 °C at 10 °C min^−1^ under nitrogen.

### 2.6. Thermal Cycling and Macroscopic Dispersion Stability Tests

Accelerated thermal cycling tests were performed on 0.25MGO to evaluate cycling reliability. Each thermal cycle consisted of melting and solidification: the sample was held at 150 °C for 10 min, cooled to 110 °C, and then held for another 10 min. This heating–cooling sequence was defined as one cycle. DSC analysis and room-temperature thermal conductivity measurements were conducted before cycling and after 100, 200, 400, and 800 cycles to evaluate the retention of phase-change and thermal transport performance. Meanwhile, 0.25GO and 0.25MGO were subjected to the same thermal cycling conditions and macroscopically observed after 0, 200, 400, and 800 cycles. For each observation, the samples were heated to 150 °C and maintained in the molten state. Macroscopic dispersion stability was evaluated by comparing the extent of phase separation and sedimentation in molten SeA–SuA.

## 3. Results

### 3.1. Theoretical Prediction of the SeA–SuA Eutectic Point

The eutectic composition and temperature of the SeA–SuA binary system were predicted using the Schrader equation [34]. Assuming ideal liquid-phase behavior, negligible mutual solubility in the solid phase, and negligible heat-capacity differences between the solid and liquid states, the relationship between the liquid-phase mole fraction of component *i* and the equilibrium temperature is expressed as:

(1) $$\ln x_{i}=-\frac{\Delta H_{\mathrm{fus},i}}{R}\left(\frac{1}{T}-\frac{1}{T_{m,i}}\right)$$

where *x_i_* is the mole fraction of component *i* in the liquid phase, *T* is the solid–liquid equilibrium temperature (K), *T_m_*_,*i*_ is the melting temperature of pure component *i* (K), Δ*H*_fus,*i*_ is the molar enthalpy of fusion of component *i* (J mol^−1^), and *R* is the universal gas constant (8.314 J mol^−1^ K^−1^).

**Figure 2:**
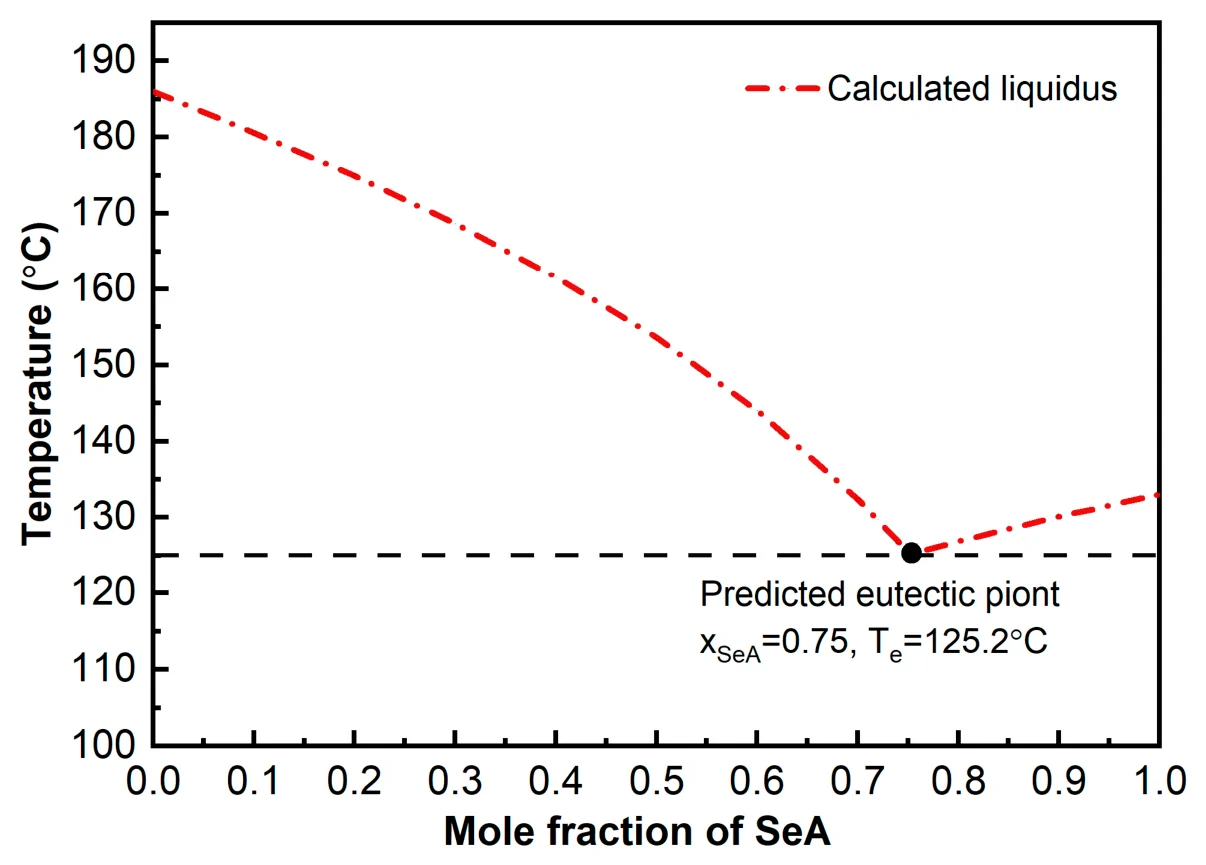
Liquidus diagram of the SeA–SuA binary system based on the Schrader equation.

The extrapolated onset melting temperatures (*T*_ei,m_), melting peak temperatures (*T*_p,m_), and melting enthalpies (Δ*H*_m_) of pure SeA and SuA were determined by DSC and are summarized in Table 2. The extrapolated onset melting temperature was determined from the intersection of the tangent to the leading edge of the melting endotherm and the extrapolated baseline. For the calculation, the extrapolated onset melting temperatures were used as (*T_m_*_,*i*_) because they more closely represent the solid–liquid equilibrium temperatures, while the mass-specific melting enthalpies were converted to molar enthalpies before substitution into Equation (1). The calculated liquidus diagram is shown in Figure 2. The intersection of the two liquidus curves defines the theoretical eutectic point at a SeA mole fraction of approximately 0.75. Accordingly, the predicted eutectic molar ratio of SeA to SuA was approximately 0.75:0.25, with a theoretical eutectic temperature of approximately 125.2 °C.

### 3.2. Phase-Change Behavior and Crystalline Structure of the SeA–SuA Mixture

**Figure 3:**
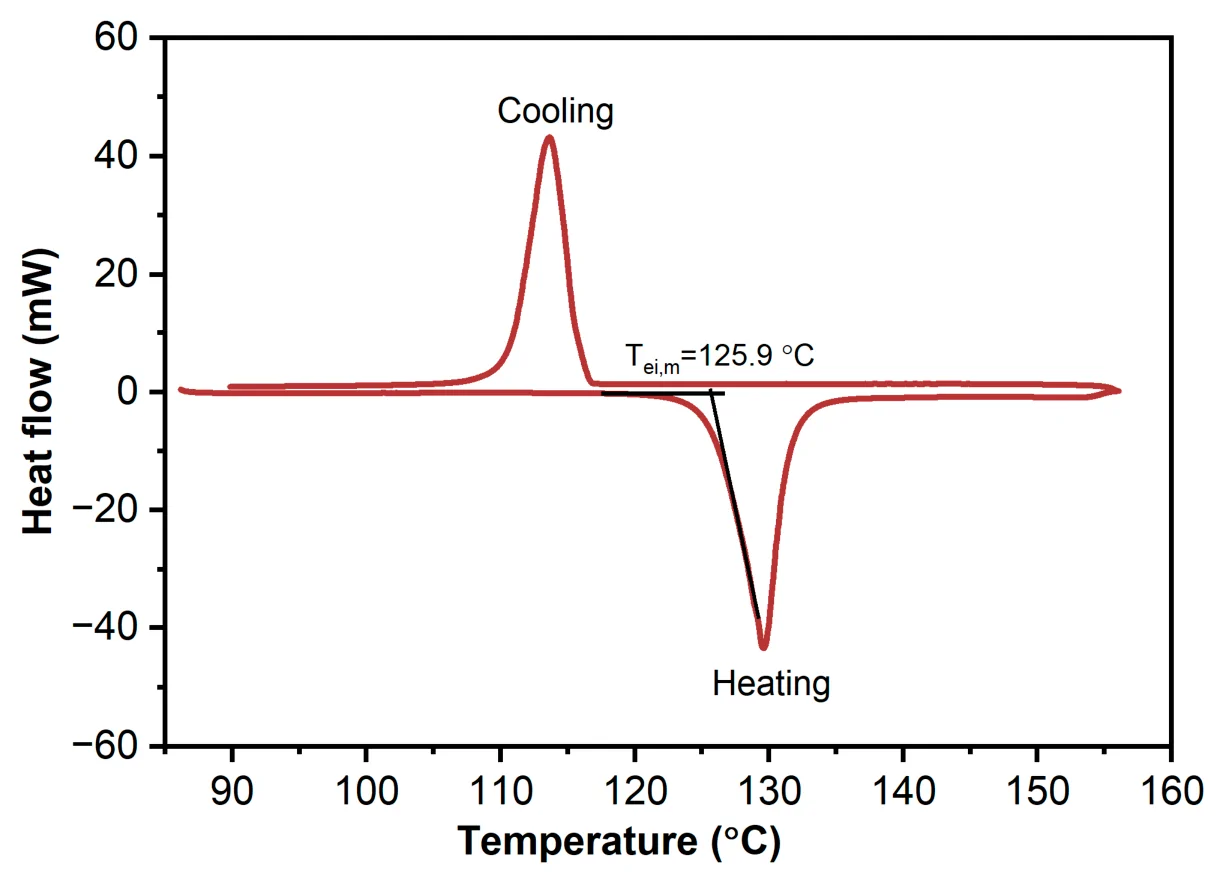
DSC heating and cooling curves of the SeA–SuA mixture at the predicted eutectic composition (SeA:SuA molar ratio = 0.75:0.25).

To evaluate the phase-change behavior at the predicted eutectic composition, DSC measurements were performed on the SeA–SuA mixture with a SeA:SuA molar ratio of 0.75:0.25. As shown in Figure 3, the sample exhibited a dominant melting endotherm during heating and a single crystallization exotherm during cooling, with no clearly resolved secondary phase-transition peaks over the investigated temperature range. This behavior suggests that melting and crystallization are each dominated by a single phase-transition event at this composition. The extrapolated onset melting temperature (*T*_ei,m_) was approximately 125.9 °C, 0.7 °C higher than the predicted eutectic temperature of 125.2 °C, showing close agreement between experiment and theory. This slight deviation may arise from the idealized assumptions of the Schrader equation and uncertainty in determining the DSC onset temperature. The mixture exhibited a melting peak temperature (*T*_p,m_) of 129.6 °C and a melting enthalpy (Δ*H*_m_) of 235.6 J g^−1^. These results indicate that the SeA–SuA mixture at a SeA:SuA molar ratio of 0.75:0.25 exhibits phase-change behavior consistent with the theoretically predicted eutectic composition.

**Figure 4:**
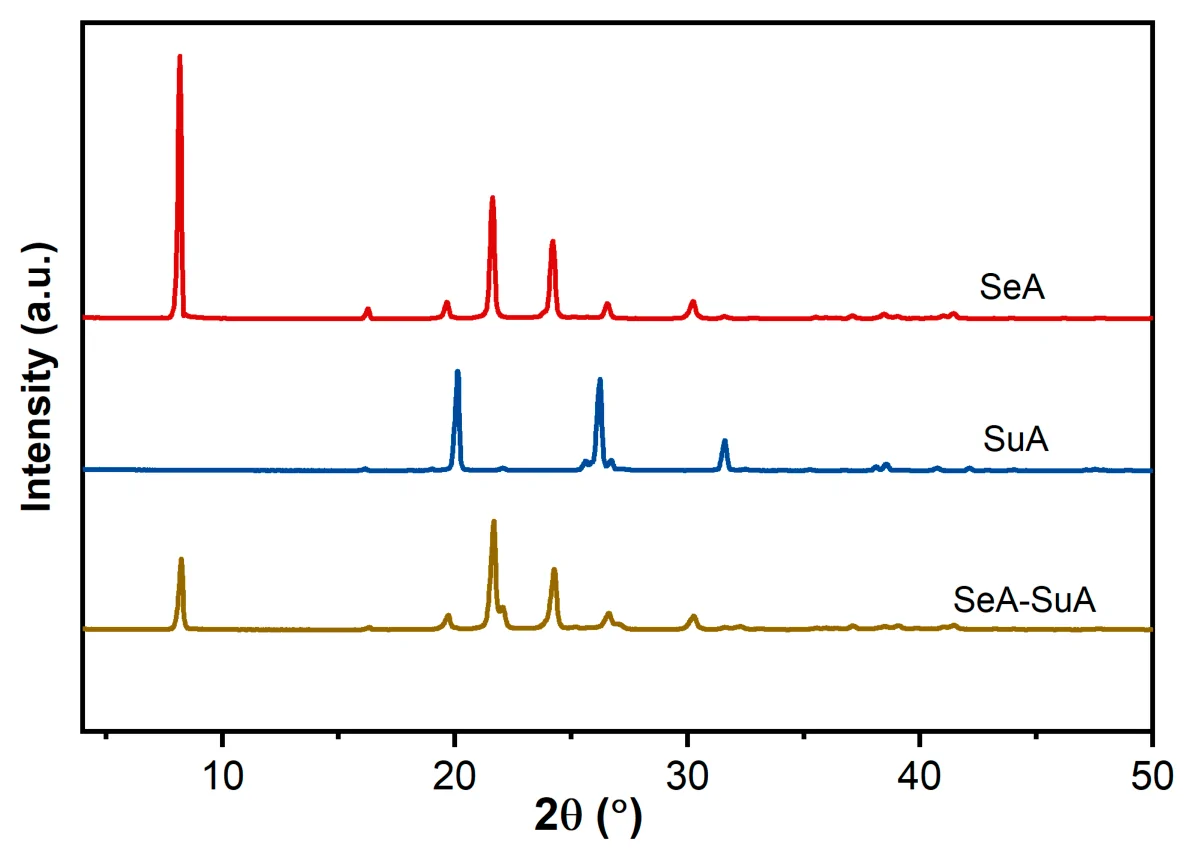
XRD patterns of SeA, SuA, and the SeA–SuA mixture.

Figure 4 presents the XRD patterns of SeA, SuA, and the SeA–SuA mixture with a SeA:SuA molar ratio of 0.75:0.25. Pure SeA and SuA exhibited distinct and sharp diffraction peaks, with their most intense peaks located at 2θ = 8.2° and 20.1°, respectively, in good agreement with values reported in the literature [35,36]. In the SeA–SuA mixture, the major diffraction peaks of both components remained observable, and no additional diffraction peaks were detected, suggesting that no new crystalline phases formed within the detection limit of XRD. This observation suggests that eutectic formation does not involve the formation of a detectable new crystalline compound. Compared with the pure components, changes in the relative intensities of several diffraction peaks were observed, with the most intense peak of the SeA–SuA mixture located at approximately 2θ = 21.7°. These intensity variations may reflect differences in component proportions, preferred orientation, and recrystallization behavior during sample solidification rather than the formation of a new crystalline phase. Overall, the XRD results indicate that SeA and SuA largely retain their respective crystalline characteristics in the mixture. Together with the single dominant phase-transition behavior observed by DSC and the close agreement between the experimentally determined extrapolated onset melting temperature and the predicted eutectic temperature, these XRD observations are consistent with the eutectic behavior of the SeA–SuA mixture at the predicted composition.

### 3.3. Chemical Characterization of PDMS-Modified GO

**Figure 5:**
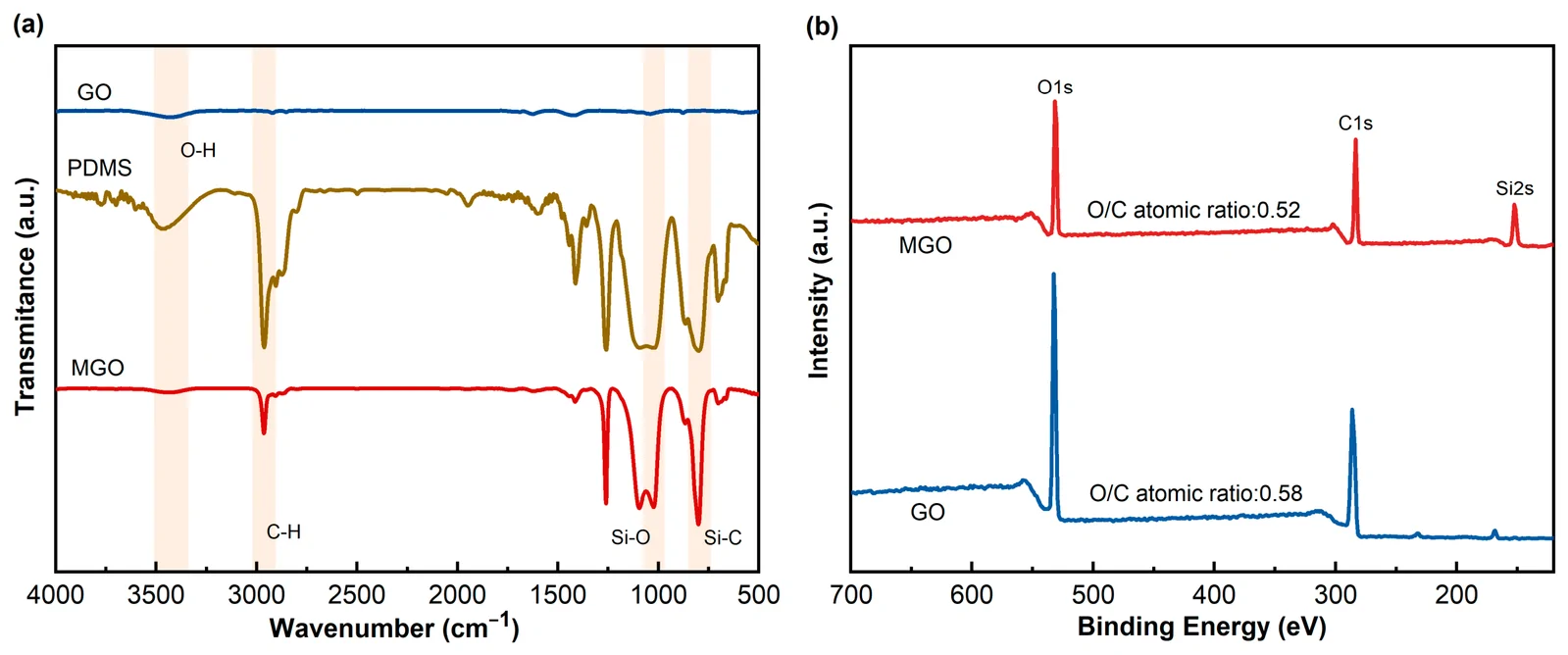
(**a**) FTIR spectra of GO, PDMS, and MGO; (**b**) XPS survey spectra of GO and MGO.

Figure 5 presents the FTIR spectra of GO, PDMS, and MGO and the XPS survey spectra of GO and MGO to examine changes in functional groups and surface elemental composition after PDMS modification. As shown in Figure 5a, GO exhibited a broad absorption band at approximately 3430 cm^−1^, which was assigned to O–H stretching vibrations, while the absorptions in the 1000–1200 cm^−1^ region were associated with vibrations of oxygen-containing groups such as C–O and C–O–C [37]. Pure PDMS showed methyl C–H stretching at approximately 2960 cm^−1^, strong Si–O–Si skeletal vibrations at 1095 and 1020 cm^−1^, and a Si–C-related band near 800 cm^−1^ [38]. Compared with pristine GO, MGO exhibited additional absorption features corresponding to PDMS, supporting the presence of PDMS-related species in the modified GO.

XPS further revealed changes in surface elemental composition after PDMS modification. As shown in Figure 5b, GO exhibited C 1s and O 1s signals, whereas an additional Si 2s signal was observed for MGO, indicating the presence of silicon-containing species on the modified GO surface. Quantitative XPS analysis showed that the O/C atomic ratio decreased from approximately 0.58 for GO to 0.52 for MGO, further indicating a change in surface elemental composition. Together, the appearance of PDMS-related absorption features in the FTIR spectrum and the additional Si signal in the XPS survey spectrum support the presence of PDMS-related species on the GO surface after modification.

### 3.4. Morphological Characterization of GO, MGO, and SeA–SuA-Based Composite PCMs

**Figure 6:**
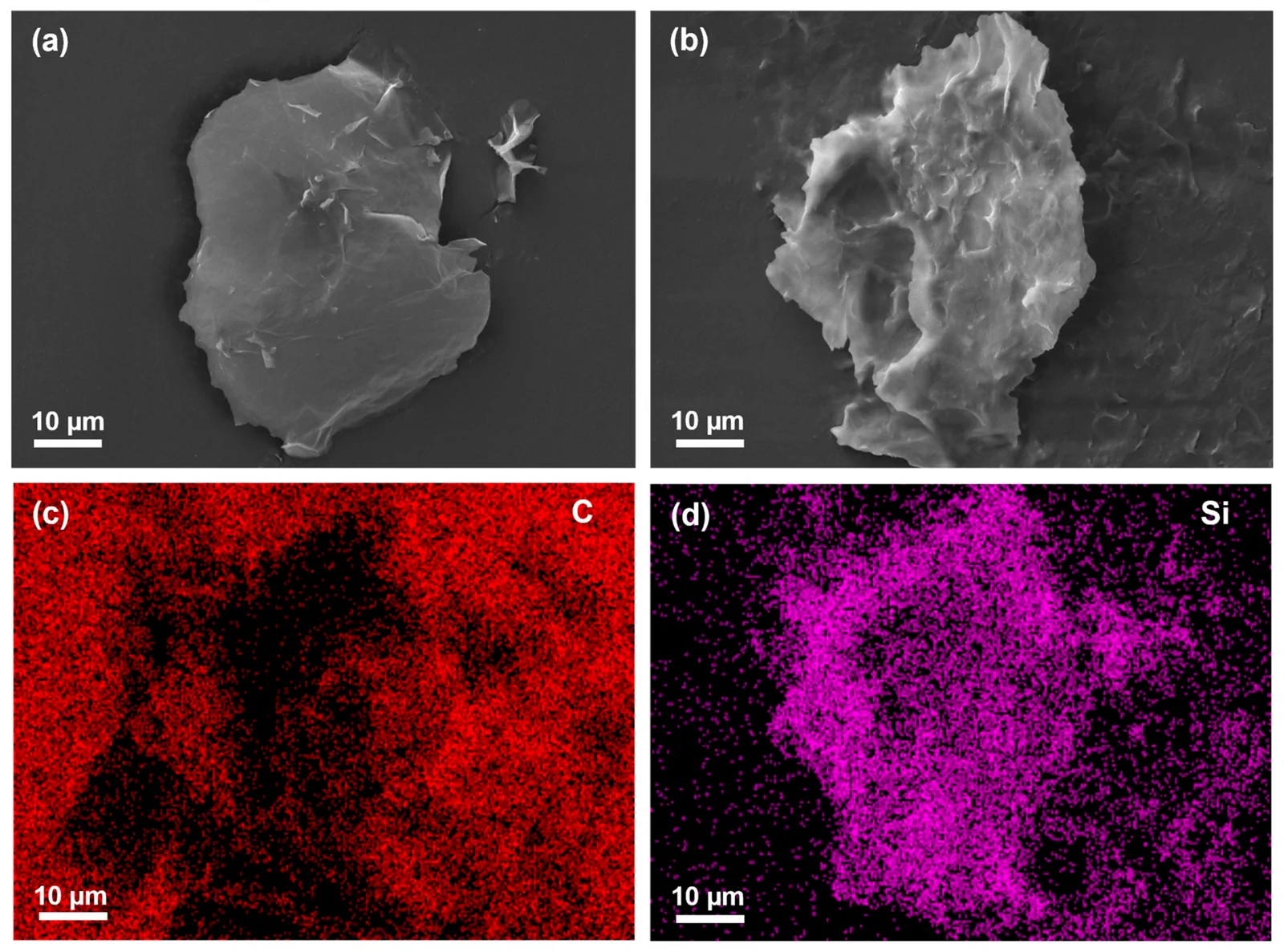
SEM images of (**a**) GO and (**b**) MGO, together with EDS elemental maps of MGO showing (**c**) C and (**d**) Si.

To further characterize the morphological changes associated with PDMS modification, GO and MGO were examined by SEM, while EDS elemental mapping was performed on MGO to investigate the spatial distribution of Si. As shown in Figure 6a, GO exhibited a sheet-like morphology with a relatively smooth surface, localized wrinkles, and curled edges. In comparison, MGO retained the sheet-like structure but displayed more pronounced surface corrugation and wrinkling, as shown in Figure 6b, suggesting morphological changes associated with PDMS modification. Figure 6c,d show the spatial distributions of C and Si, respectively. The Si signal was broadly distributed across the sheet-like regions of MGO. Together with the FTIR and XPS results discussed above, these observations further support the presence of PDMS-related species on the GO surface after modification.

**Figure 7:**
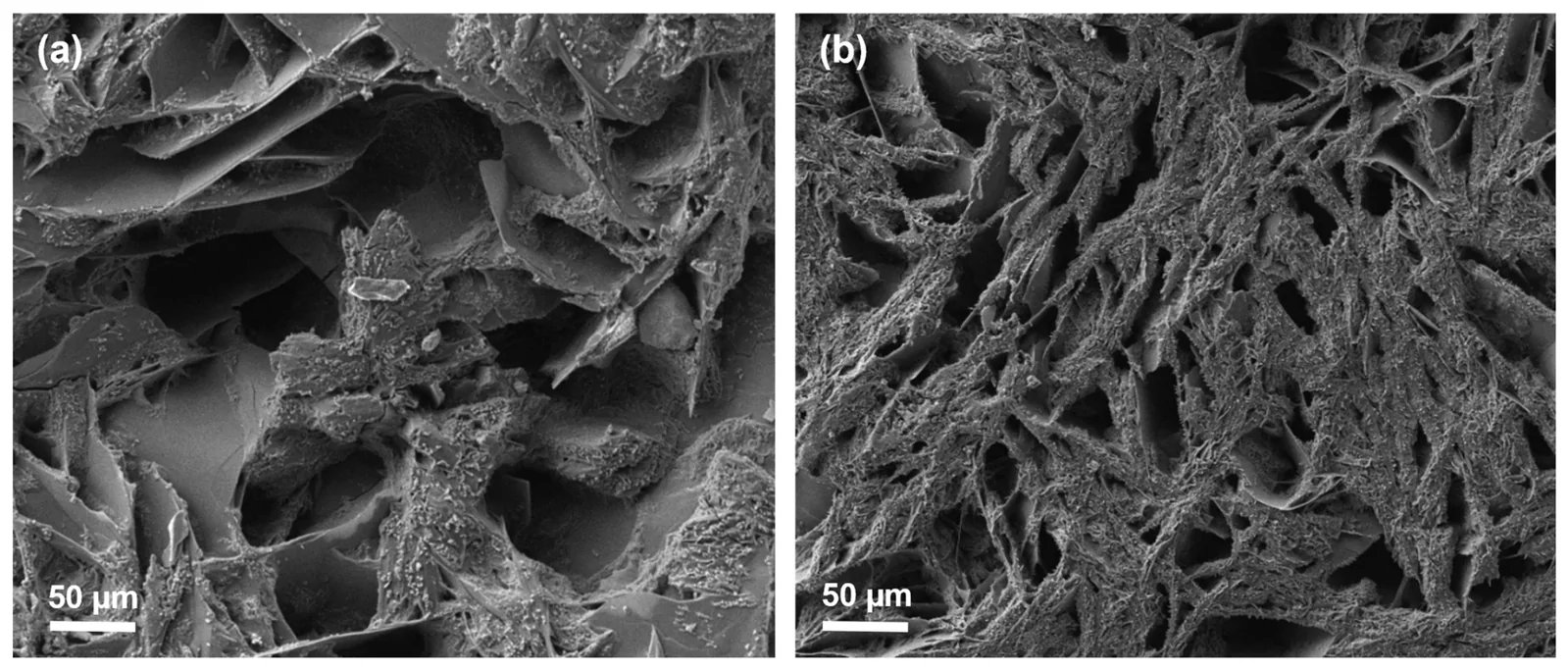
Cross-sectional SEM images of (**a**) 0.25GO and (**b**) 0.25MGO composite PCMs.

To examine the effect of PDMS modification on filler distribution and filler–matrix contact within the SeA–SuA matrix, the cross-sectional morphologies of the 0.25GO and 0.25MGO composite PCMs were compared. As shown in Figure 7a, relatively large sheet-like structures, localized stacking, and pronounced voids were observed in 0.25GO, with distinct morphological boundaries between some sheet-like regions and the surrounding matrix. In contrast, 0.25MGO exhibited a finer and more interconnected microstructure, as shown in Figure 7b. No large-scale sheet stacking comparable to that observed in 0.25GO was evident within the examined region, and the morphological transition between the filler and surrounding matrix appeared more continuous. These observations suggest that PDMS surface modification may reduce sheet aggregation and improve filler distribution and filler–matrix contact within the SeA–SuA matrix. Such improved contact may reduce thermal transport limitations associated with localized aggregation and interfacial discontinuities, thereby providing a possible microstructural basis for the improved thermal transport behavior discussed subsequently.

### 3.5. Thermal Properties of SeA–SuA and SeA–SuA-Based Composite PCMs

**Figure 8:**
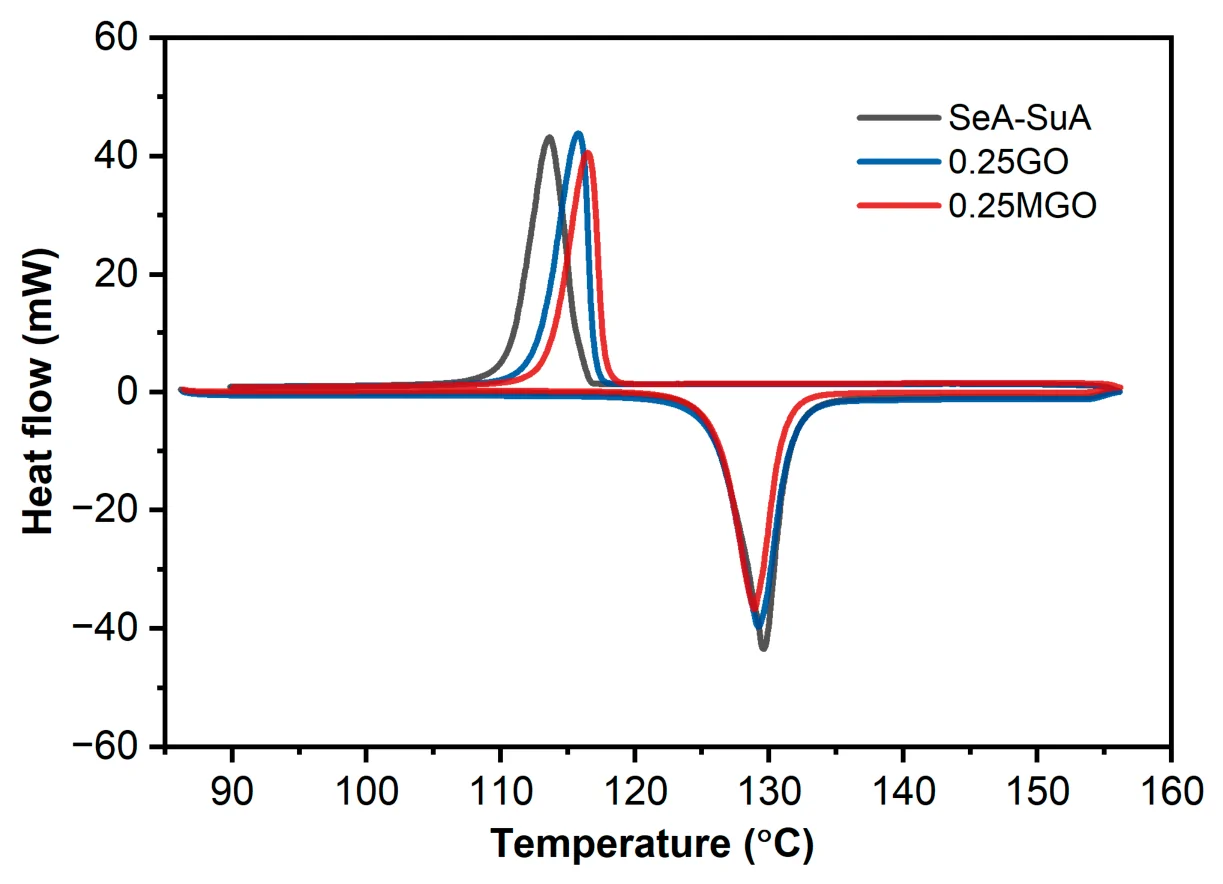
DSC heating and cooling curves of SeA–SuA, 0.25GO, and 0.25MGO.

To evaluate the effect of PDMS modification of GO on the phase-change behavior of the composite PCMs, DSC measurements were performed on SeA–SuA, 0.25GO, and 0.25MGO, as shown in Figure 8 and Table 3. Compared with 0.25GO, the crystallization peak temperature (*T*_p,c_) of 0.25MGO increased from 115.7 to 116.5 °C, whereas the melting peak temperature (*T*_p,m_) changed only slightly. Consequently, the degree of supercooling (Δ*T*) decreased from 13.6 to 12.4 °C. The higher crystallization peak temperature and lower degree of supercooling suggest that the incorporation of MGO may facilitate the crystallization of SeA–SuA. In conjunction with the morphological observations discussed above, this behavior may be associated with reduced sheet aggregation and improved filler–matrix contact after PDMS modification. In addition, the two-dimensional filler surfaces may provide heterogeneous nucleation sites, and their improved dispersion in 0.25MGO may increase the effective contact between these sites and the PCM matrix.

Compared with 0.25GO, 0.25MGO also exhibited improved latent heat retention. The melting enthalpy of 0.25MGO was 234.8 J g^−1^, 14.3 J g^−1^ higher than that of 0.25GO (220.5 J g^−1^), corresponding to a retention of approximately 99.7% relative to SeA–SuA. Because GO and MGO were incorporated at the same nominal filler loading, the difference in latent heat between the two composites cannot be explained by the dilution effect of the non-phase-changing filler alone. For comparison, simple mass dilution by a 0.25 wt% non-phase-changing filler would give an expected melting enthalpy of approximately 235.0 J g^−1^, which is close to the measured value of 234.8 J g^−1^ for 0.25MGO. Instead, the difference may be associated with changes in filler distribution and filler–matrix contact following PDMS modification. Reduced localized aggregation may lessen the disruption of PCM crystallization associated with aggregated GO sheets. For 0.25MGO, the crystallization enthalpy (226.9 J g^−1^) was approximately 96.6% of its melting enthalpy (234.8 J g^−1^), indicating comparable latent heat absorption and release under the DSC testing conditions. However, this ratio does not represent the overall energy storage conversion efficiency, which requires system-level measurements of heat input and recovery accounting for sensible heat and thermal losses. Overall, compared with 0.25GO, the 0.25MGO composite exhibited better latent heat retention and a lower degree of supercooling.

**Figure 9:**
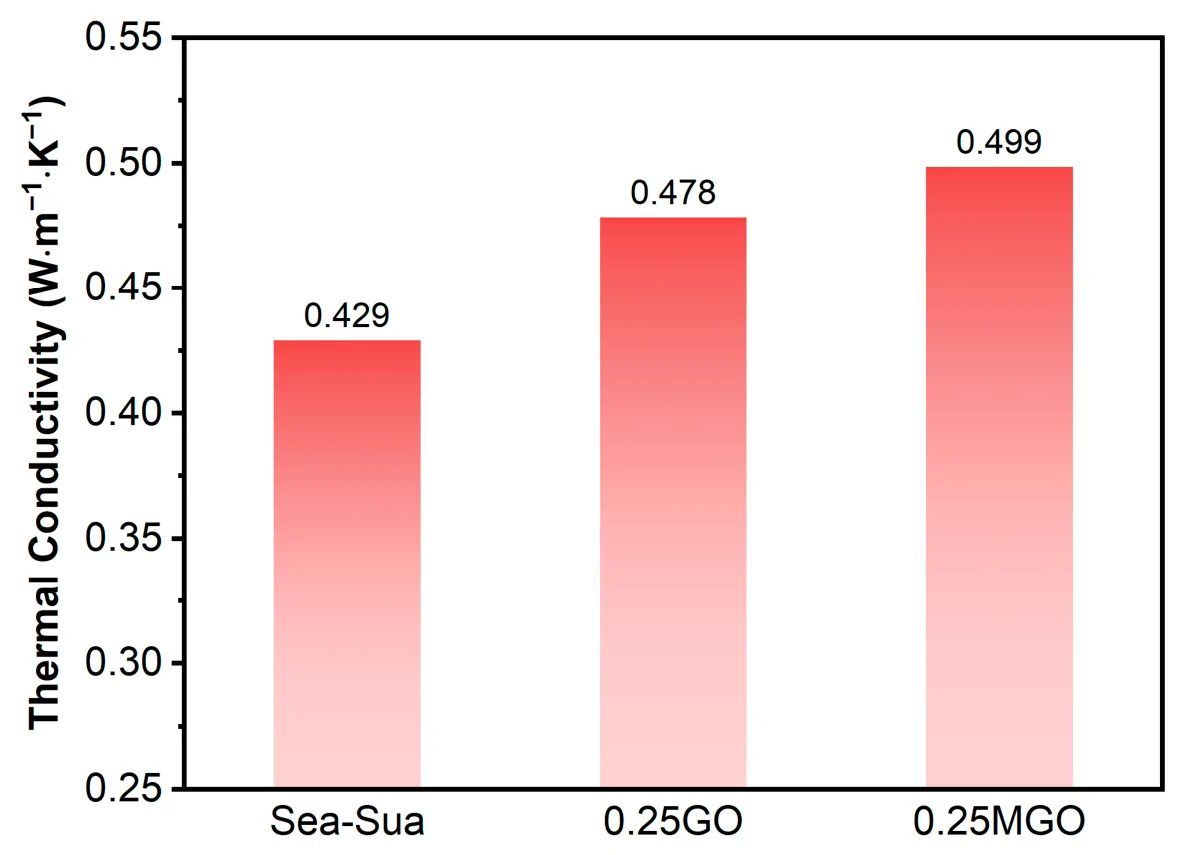
Room-temperature thermal conductivity of SeA–SuA, 0.25GO, and 0.25MGO.

In addition to latent heat storage, thermal conductivity is an important parameter governing thermal transport in composite PCMs. As shown in Figure 9, the room-temperature thermal conductivity of SeA–SuA was 0.429 W m^−1^ K^−1^. The thermal conductivity increased to 0.478 W m^−1^ K^−1^ for 0.25GO and further to 0.499 W m^−1^ K^−1^ for 0.25MGO. At the same nominal filler loading, the thermal conductivity of 0.25MGO was 4.4% higher than that of 0.25GO and approximately 16.3% higher than that of SeA–SuA. The higher thermal conductivity of 0.25MGO may be associated with reduced sheet aggregation and improved filler–matrix contact following PDMS modification. These features may reduce interruptions in heat-transfer pathways and facilitate more effective thermal transport through the composite, consistent with the SEM observations discussed above.

Table 4 compares the thermophysical properties of 0.25MGO with representative PCMs operating in a similar temperature range. The 0.25MGO composite exhibited a relatively high melting enthalpy of 234.8 J g^−1^ and a 16.3% increase in thermal conductivity relative to SeA–SuA at a nominal filler loading of only 0.25 wt%. Although some graphite-supported composites exhibited higher thermal conductivity, the present results demonstrate the potential of PDMS-modified GO to improve thermal transport while maintaining high latent heat storage capacity.

**Figure 10:**
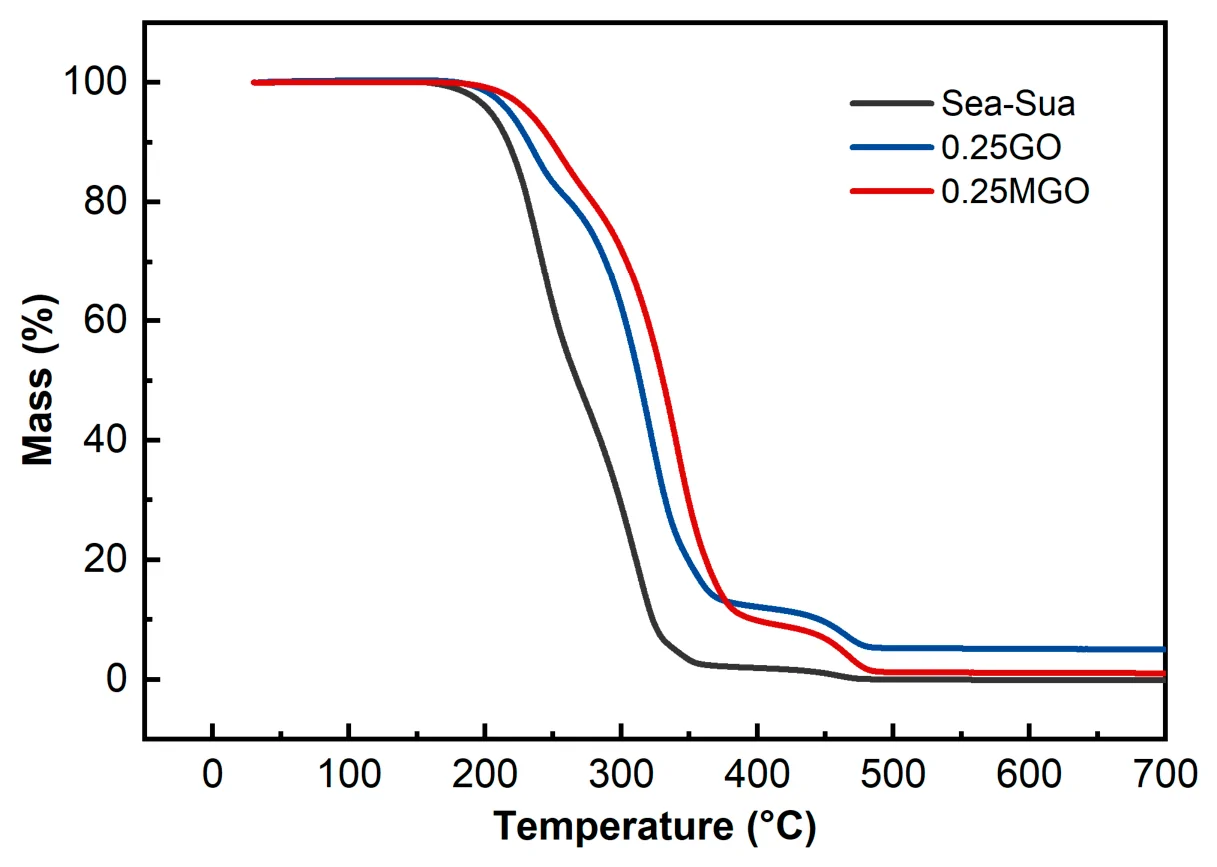
TG curves of SeA–SuA, 0.25GO, and 0.25MGO.

Thermogravimetric analysis was also performed to evaluate the thermal stability and mass-loss behavior of SeA–SuA, 0.25GO, and 0.25MGO during heating. As shown in Figure 10, no pronounced mass loss was observed below approximately 180 °C for any of the samples, indicating minimal mass loss at the phase-change temperature of approximately 129 °C under the dynamic TGA conditions. With increasing temperature, SeA–SuA exhibited substantial mass loss, with the main degradation region spanning approximately 200–350 °C. The TG curves of both composites shifted toward higher temperatures, and 0.25MGO showed greater mass retention than SeA–SuA over the main degradation region, suggesting enhanced resistance to thermal decomposition under dynamic heating conditions. Overall, the 0.25MGO composite combined enhanced thermal conductivity with improved resistance to thermal decomposition under the tested dynamic heating conditions.

### 3.6. Thermal Cycling Performance and Macroscopic Dispersion Stability

**Figure 11:**
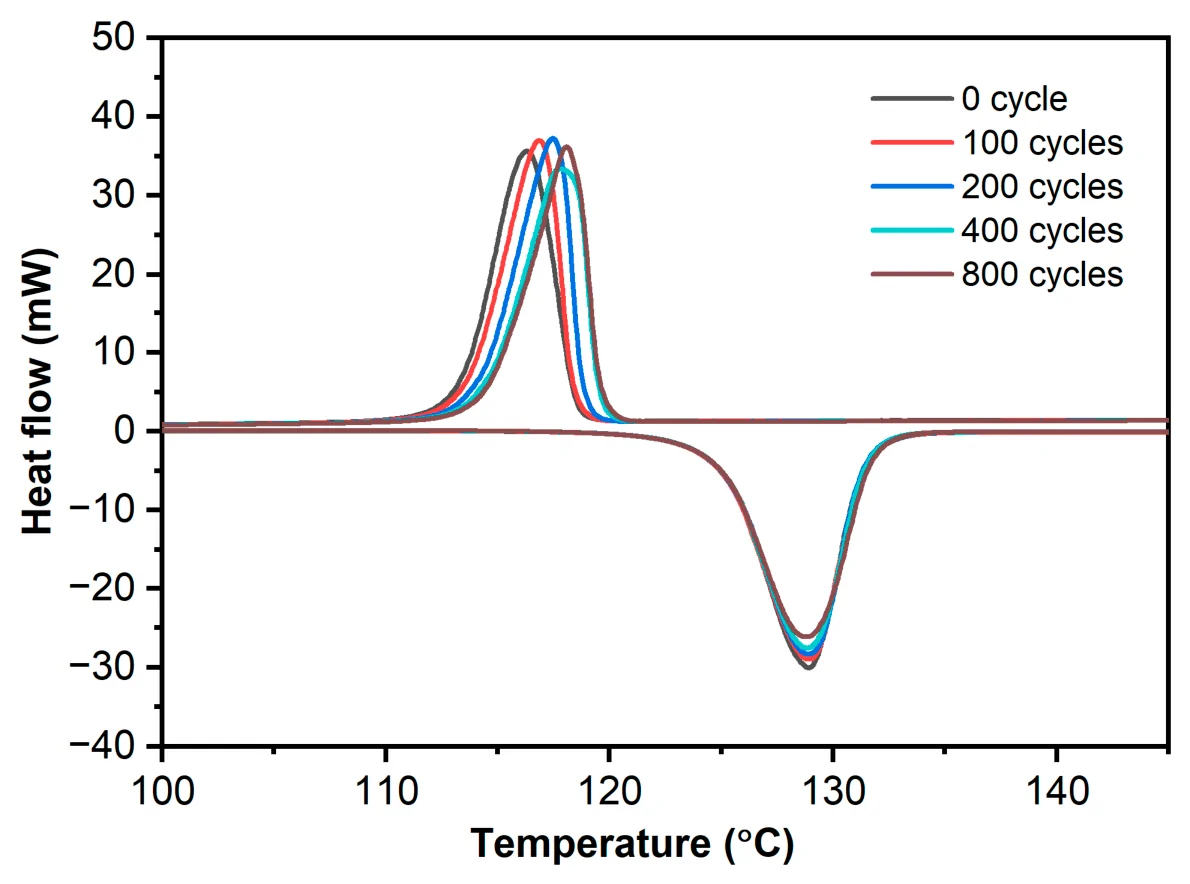
DSC heating and cooling curves of the 0.25MGO composite PCM after different numbers of thermal cycles.

The ability of a PCM to retain its thermal performance during repeated melting and solidification is an important indicator of its long-term applicability. Therefore, thermal cycling tests were performed on the 0.25MGO composite PCM, with DSC analysis and room-temperature thermal conductivity measurements conducted before cycling and after 100, 200, 400, and 800 cycles. The DSC curves are shown in Figure 11, and the corresponding thermal properties are summarized in Table 5. Across the investigated cycle numbers, 0.25MGO retained well-defined melting and crystallization peaks, while the overall DSC curve shapes remained similar, indicating that its reversible solid–liquid phase-change behavior was largely preserved. The melting peak temperature (*T*_p,m_) remained nearly unchanged, whereas the crystallization peak temperature (*T*_p,c_) gradually increased from 116.5 °C before cycling to 118.1 °C after 800 cycles. Accordingly, the degree of supercooling decreased from 12.4 to 10.7 °C after 800 thermal cycles.

The phase-change enthalpies of 0.25MGO gradually decreased with increasing cycle number. After 800 thermal cycles, the melting enthalpy (Δ*H*_m_) decreased from 234.8 to 223.6 J g^−1^, corresponding to a retention of approximately 95.2%. The crystallization enthalpy (Δ*H*_c_) decreased from 226.9 to 219.5 J g^−1^, corresponding to a retention of approximately 96.7%. The room-temperature thermal conductivity decreased from 0.499 to 0.438 W m^−1^ K^−1^, corresponding to a retention of approximately 87.8%. The larger relative decrease in thermal conductivity compared with phase-change enthalpy suggests that repeated melting and solidification may alter the spatial distribution of the filler, filler–filler contacts, or filler–matrix interfaces, thereby affecting effective thermal transport pathways within the composite. Overall, 0.25MGO retained an essentially stable melting peak temperature and approximately 95.2% of its initial melting enthalpy after 800 cycles, indicating that its phase-change performance was largely retained.

**Figure 12:**
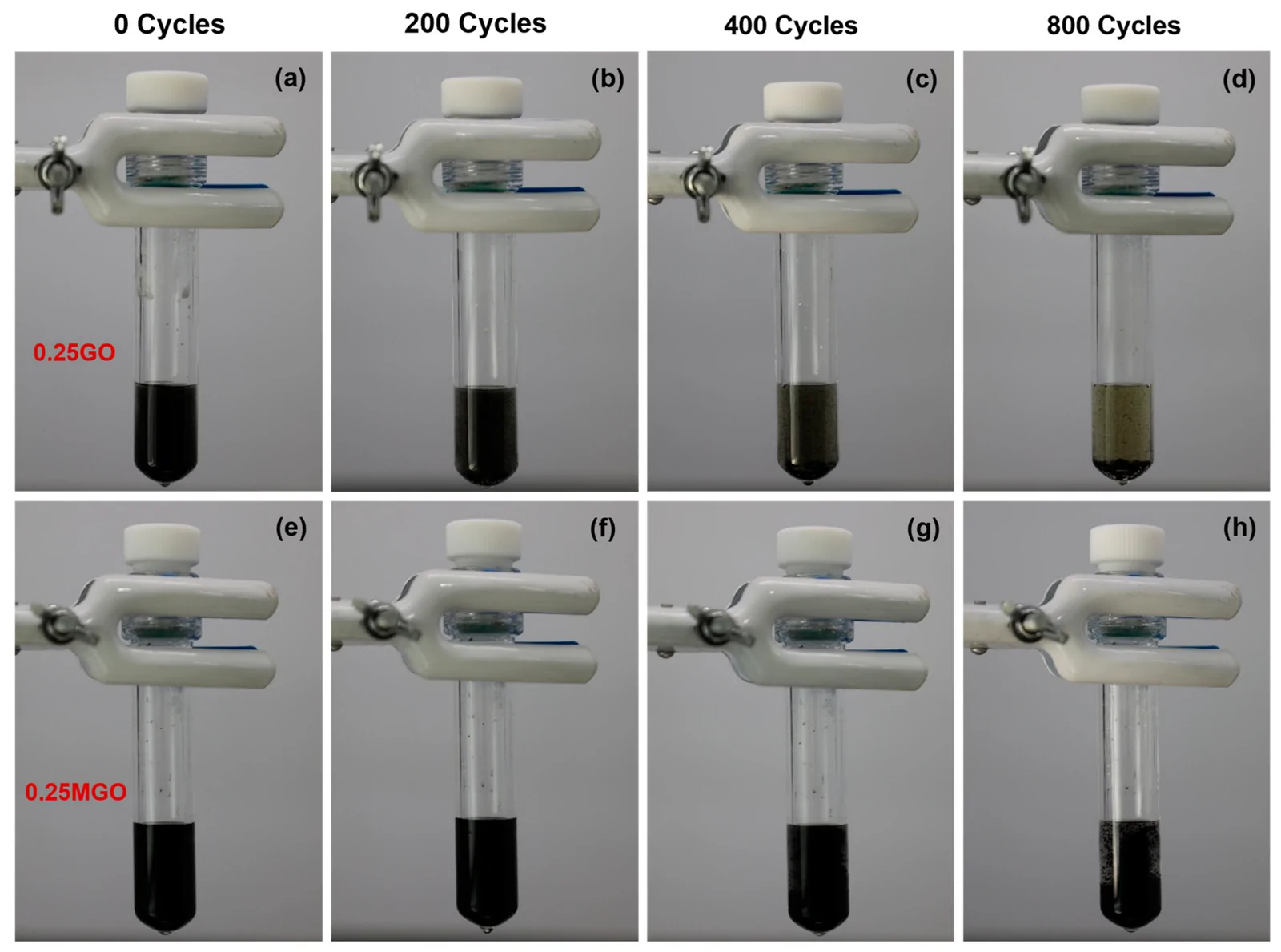
Macroscopic dispersion states of 0.25GO and 0.25MGO in the molten state after different numbers of thermal cycles: (**a**,**e**) 0 cycles; (**b**,**f**) 200 cycles; (**c**,**g**) 400 cycles; and (**d**,**h**) 800 cycles.

In addition to phase-change performance, macroscopic sedimentation behavior provides useful information on filler dispersion stability in composite PCMs. To compare the effect of PDMS surface modification on the dispersion behavior of GO in the SeA–SuA eutectic matrix, 0.25GO and 0.25MGO were subjected to identical thermal cycling conditions and macroscopically examined in the molten state at 150 °C after 0, 200, 400, and 800 cycles. As shown in Figure 12, 0.25GO exhibited progressively more pronounced phase separation and sedimentation with increasing cycle number, whereas 0.25MGO maintained comparatively better macroscopic dispersion under the same conditions. This difference may be associated with changes in the surface characteristics of GO and steric hindrance introduced by PDMS modification, which could reduce direct contact and restacking between adjacent sheets. These observations are consistent with the reduced large-scale aggregation observed by SEM and suggest that PDMS modification may improve the dispersion stability of MGO in the SeA–SuA matrix. Improved dispersion may help maintain more uniform filler–matrix contact and reduce the formation of locally filler-depleted regions during repeated melting and solidification, thereby contributing to the preservation of effective thermal transport pathways. Nevertheless, the gradual decrease in thermal conductivity during cycling suggests that the effective thermal transport network is not fully stable. Repeated phase transitions may still induce partial redistribution of MGO sheets or changes in filler–filler and filler–matrix contacts, leading to progressive deterioration of thermal transport. In practical TES systems, filler redistribution or sedimentation during prolonged cycling could lead to nonuniform heat transfer and reduced charging/discharging performance. However, without tests beyond 800 cycles or a quantitative degradation model, the long-term evolution of thermal conductivity remains uncertain. Therefore, the improved macroscopic dispersion stability of 0.25MGO helps explain its observed cycling stability, while further stabilization of the conductive network remains important for long-term application.

## 4. Conclusions

This study combined eutectic composition regulation with PDMS modification of GO to develop a SeA–SuA/MGO composite PCM for medium-temperature thermal energy storage. The Schrader equation predicted a eutectic SeA:SuA molar ratio of 0.75:0.25 and a eutectic temperature of 125.2 °C, with the latter in good agreement with the experimentally determined extrapolated onset melting temperature of 125.9 °C. At an MGO loading of 0.25 wt%, the composite exhibited a melting peak temperature of 128.9 °C, a melting enthalpy of 234.8 J g^−1^, and a thermal conductivity of 0.499 W m^−1^ K^−1^. Compared with 0.25GO at the same nominal filler loading, 0.25MGO exhibited a lower degree of supercooling, better latent heat retention, and higher thermal conductivity. Together with the morphological and macroscopic dispersion observations, these results suggest that PDMS modification may improve filler dispersion and filler–matrix contact within the SeA–SuA matrix. After 800 thermal cycles, 0.25MGO retained approximately 95.2% of its initial melting enthalpy, indicating good retention of phase-change performance. Its thermal conductivity retention was lower, at approximately 87.8%, indicating that thermal transport stability during cycling still requires further improvement. The environmental impacts, potential toxicity, and mechanical properties of the composite were not evaluated in this study and warrant further investigation. Overall, the combination of eutectic composition regulation and PDMS modification of GO provides a feasible strategy for designing medium-temperature composite PCMs with a suitable phase-change temperature and a favorable balance among latent heat retention, thermal transport, and cycling performance.

## Figures and Tables

**Table 1:** Sample codes and nominal compositions of the SeA–SuA-based PCMs.

Sample Code	SeA–SuA (wt%)	GO (wt%)	MGO (wt%)
SeA–SuA	100.00	0.00	0.00
0.25GO	99.75	0.25	0.00
0.25MGO	99.75	0.00	0.25

Note: The compositions are nominal mass percentages calculated from the feed masses relative to the total mass of all components. The SeA:SuA molar ratio in the eutectic matrix was 0.75:0.25.

**Table 2:** DSC-derived melting parameters of SeA and SuA used in the eutectic calculation.

Sample	*T*_ei,m_ (°C)	*T*_p,m_ (°C)	Δ*H*_m_ (J g^−1^)
SeA	133	135	245
SuA	186	190	295

**Table 3:** Phase-change parameters of SeA–SuA, 0.25GO, and 0.25MGO.

Sample	*T*_p,m_ (°C)	*T*_p,c_ (°C)	Δ*T* (°C)	Δ*H*_m_ (J g^−1^)	Δ*H*_c_ (J g^−1^)
SeA–SuA	129.6	113.6	16.0	235.6	228.1
0.25GO	129.3	115.7	13.6	220.5	211.3
0.25MGO	128.9	116.5	12.4	234.8	226.9

Note: The degree of supercooling was calculated as Δ*T* = *T*_p,m_ − *T*_p,c_.

**Table 4:** Comparison of thermophysical properties of representative PCMs for medium-temperature thermal energy storage.

PCM System	*T*_p,m_ (°C)	Δ*H*_m_ (J g^−1^)	λ (W m^−1^ K^−1^)	Reference
SeA–SuA	129.6	235.6	0.429	This work
0.25GO	129.3	220.5	0.478	This work
0.25MGO	128.9	234.8	0.499	This work
SeA/EG	128.0	187.0	5.35	[39]
SeA/AA/EG/NaAlg	117.3	194.6	0.32	[40]
MgCl_2_·6H_2_O	115.1	166.9	0.70	[41]

Note: AA, adipic acid; EG, expanded graphite; NaAlg, sodium alginate. Phase-change temperature definitions and measurement conditions vary among studies.

**Table 5:** Changes in the thermal properties of the 0.25MGO composite PCM during thermal cycling.

Cycles	*T*_p,m_ (°C)	*T*_p,c_ (°C)	Δ*H*_m_ (J g^−1^)	Δ*H*_c_ (J g^−1^)	λ (W m^−1^ K^−1^)
0	128.9	116.5	234.8	226.9	0.499
100	128.9	116.9	230.3	225.8	0.475
200	128.9	117.5	228.2	224.1	0.462
400	128.8	117.8	227.3	222.1	0.451
800	128.8	118.1	223.6	219.5	0.438

## Data Availability

The original contributions presented in this study are included in the article. Further inquiries can be directed to the corresponding authors.

## Abbreviations

The following abbreviations are used in this manuscript:

DSC	Differential scanning calorimetry
EDS	Energy-dispersive X-ray spectroscopy
FTIR	Fourier-transform infrared spectroscopy
GO	Graphene oxide
HP–ORC	Heat pump–organic Rankine cycle
MGO	PDMS-modified graphene oxide
OA	Oxalic acid dihydrate
PCM	Phase change material
PDMS	Polydimethylsiloxane
SeA	Sebacic acid
SEM	Scanning electron microscopy
SuA	Succinic acid
TES	Thermal energy storage
TG	Thermogravimetry
TGA	Thermogravimetric analysis
TPS	Transient plane source
XPS	X-ray photoelectron spectroscopy
XRD	X-ray diffraction
